# The Sleep Patterns of Children and Adolescents with Chronic Conditions and Their Families: An Integrative Literature Review

**DOI:** 10.3390/children11020207

**Published:** 2024-02-06

**Authors:** Welker da Silva Xavier, Madalena Paulos Abreu, Michelle Darezzo Rodrigues Nunes, Fernanda Machado Silva-Rodrigues, Liliane Faria da Silva, Barbara Bertolossi Marta de Araújo, Paula Saud De Bortoli, Rhyquelle Rhibna Neris, Lucila Castanheira Nascimento

**Affiliations:** 1 Department of Maternal-Infant Nursing, Nursing Faculty, Rio de Janeiro State University, Rio de Janeiro 20551-030, RJ, Brazil; welker_xavier@hotmail.com (W.d.S.X.); madapalins@gmail.com (M.P.A.); betabertolossiuerj@gmail.com (B.B.M.d.A.); 2 Maternal-Infant and Psychiatric Nursing Department, School of Nursing, University of Sao Paulo, São Paulo 05403-000, SP, Brazil; fermachado@usp.br; 3 Maternal-Infant and Psychiatric Nursing Department, Aurora de Afonso Costa School of Nursing, Fluminense Federal University, Niteroi 22020-091, RJ, Brazil; lilianefaria@id.uff.br; 4 Maternal-Infant and Public Health Nursing, Ribeirão Preto College of Nursing, University of São Paulo, Ribeirão Preto 14040-902, SP, Brazil; paula.bortoli@usp.br (P.S.D.B.); rhyquelle@usp.br (R.R.N.); lucila@eerp.usp.br (L.C.N.)

**Keywords:** child, adolescent, family, chronic disease, sleep–wake disorders

## Abstract

Sleep is of vital necessity for health, and it has a restorative and protective function for children and adolescents with chronic conditions and their families. The purpose of this study was to identify the scientific production on sleep patterns in children and adolescents with chronic conditions and their families. This integrative review was conducted between March and June 2022 using the databases of MEDLINE, Web of Science, CINAHL and PsycINFO. The articles included were original papers published between January 2007 and mid-2022. Excluded were review studies that did not evaluate sleep and whose participants did not have chronic conditions or were not children, adolescents and/or their families. The searches returned 814 abstracts. After exclusions, 47 studies were selected to be read in full; of these, 29 were selected and were grouped empirically into four categories: major alterations in the sleep patterns of children and adolescents with chronic conditions; the relationship between sleep disorders and symptoms in children and adolescents with chronic conditions; the impaired sleep patterns of families of children and adolescents with chronic conditions; and sleep alterations and their relationship with other problems in families of children and adolescents with chronic conditions. All studies showed sleep pattern impairment in children and adolescents with chronic conditions as well as their families.

## 1. Introduction

Chronic conditions are becoming more prevalent globally, affecting not only adults but also children and young people [1]. These conditions often lead to significant changes in daily routines and increased reliance on various technologies and medications. The resultant side effects, along with the challenge of managing multiple symptoms, can prolong hospital stays. A particularly notable impact of these factors is the prevalence of sleep disturbances, which can significantly impair the quality of life of both young patients and their families. This complex interplay of health challenges underscores the need for comprehensive care and support systems [2].

Some of the main chronic conditions that affect children include asthma, obesity, behavioral and learning problems [3], allergies, kidney and rheumatological diseases, cancer, cystic fibrosis, diabetes, sickle cell disease, Down’s syndrome, and congenital malformations [4].

Sleep meets a basic human need in maintaining wellbeing. Especially in children and adolescents, recurrent sleep interruptions can interfere with growth, development, learning and health [2,5], while in adults, they can lead to psychological disorders, cognitive deficit, metabolic alterations, obesity, hypertension, and diabetes [6,7].

In children with chronic conditions, the major sleep disorders are impaired sleep quality and hygiene, and difficulty falling asleep. In hospital, children also tend to sleep less, because of diagnosis- and care-related factors [8]. Studies show that sleep disorders affect one in four children [9]. The recognition and efficient management of these disorders are important to prevent impairments in physical, cognitive, emotional, neurobehavioral, and social development [9]. Furthermore, when considering the permanent nature of chronic conditions, added to the use of life support technologies and various medications and, consequently, living with numerous symptoms, adverse effects, and the need for frequent and prolonged hospitalizations, they become a frequent problem in sleep patterns, negatively influencing the quality of life of children and adolescents [2].

Scientific evidence points to the susceptibility of sleep disorders in this population, with emphasis on the difficulties in initiating and maintaining sleep, respiratory problems associated with sleep, daytime drowsiness, parasomnia and irregular rhythms of sleep and wakefulness [10].

Major sleep alterations in adults include insomnia, breathing-related sleep disorders, central disorders of hypersomnolence, parasomnias, Circadian rhythm sleep disorders, and sleep-related movement disorders [11]. The consequences of experiencing sleep disorders also include intensified symptoms such as pain, fatigue, behavioral problems, anxiety, depression, aggression, and withdrawal [12].

A child’s illness and hospitalization also alter their family’s routines, leading to burnout and physical, mental, and social problems. Providing 24 h care also leads to modifications in families’ sleep patterns, placing them at risk of health problems and threatening their ability to provide care [13].

It is noteworthy that the experience of sleep-disorder symptoms in children and adolescents with chronic conditions has a high correlation with sleep deprivation in their family members. A study carried out with children with neurological conditions, in addition to finding the presence of this symptom, showed that the more severe the child’s condition, the more family members needed to provide care at night, which results in physical, mental, and social wear and tear, and also affects parents’ working capacity. Thus, more than 50% of them suffered from a sleep disorder, such as prolonged sleep latency and reduced duration [10].

In light of these problems, this article aimed to identify the scientific production in the literature on sleep patterns in children and adolescents with chronic health conditions and their families.

## 2. Materials and Methods

An integrative review [14] was carried out in the following stages: identifying the topic and selecting the review question; establishing inclusion and exclusion criteria; specifying the information to be extracted and categorizing the studies; evaluating the studies included; interpreting the findings; and synthesizing the knowledge [15]. This integrative review approach was chosen for its alignment with EBP methodologies. This method stands out among review methodologies due to its comprehensive scope, allowing for the inclusion of a broad range of research types, such as experimental and quasi-experimental studies. Integrative reviews enable a more complete understanding of the subject matter, leading to greater depth and breadth in the conclusions [15].

The review question was “What are sleep patterns like in children and adolescents with chronic conditions and their families?”. This was formulated using the PICo acronym [16], with “P” corresponding to the population/problem (children, adolescents and their families); “I”, to the phenomenon of interest (sleep pattern); and “C”, to the context (chronic condition).

Data were collected between March and July 2022. The data bases searched were MEDLINE^®^ (via PubMed^®^), Web of Science, Cumulative Index of Nursing and Allied Health (CINAHL), and PSYCInfo. The standard search strategy, using controlled descriptors and alternative terms identified in Medical Subject Headings (MESH), was: (Child OR Children OR Adolescent OR Adolescents OR Teen OR Teenager OR Youth) AND (“Chronic Disease” OR “Chronic Diseases” OR “Disease, Chronic” OR “Chronic Illness” OR “Illness, Chronic” OR “Chronic condition” OR “Chronic Conditions”) AND (Sleep OR “Sleep patterns” OR “Sleep Habits” OR “Sleep Wake Disorders” OR “Sleep Wake Disorder” OR “Wake Disorders, Sleep” OR “Sleep Disorders” OR “Sleep Disorder”) AND (Caregivers OR Caregiver OR Parents OR Family OR “Family Caregiver” OR Families OR “Family Members”). Also, the terms “Child, Preschool”, Infant and Adolescence, found in the CINAHL headings, were added to the search of the CINAHL base, and the terms “Child Health” and “Adolescent Health”, found in the APA thesaurus, were added to the search of the PSYCInfo base. Other articles were included by active searching.

Original research studies published between January 2007 and June 2022 were included, considering the most recent publication of the American Academy of Sleep guide to using actigraphy, which stipulated in 2007 that “actigraphy is indicated to delineate sleep patterns and document responses to treatment in normal infants and children and in special pediatric populations” [17]. The criteria excluded review studies, editorials and abstracts that did not evaluate sleep, whose participants had no chronic condition, or which were not conducted with children, adolescents or their families.

In addressing the impact of chronic conditions on children and adolescents, significant alterations in their sleep patterns are observed, and this can also be seen in their families where their sleep patterns are affected by the demands of chronic care. This area, particularly under-researched in the Brazilian context, represents a crucial knowledge gap. The current study, part of a broader project, assesses these sleep patterns using wrist actigraphy. This method is especially relevant where traditional polysomnography proves challenging and is thus a preferred method of sleep disorder evaluation [17].

The American Academy of Sleep Medicine (AASM), in its guide on practical parameters for the use of actigraphy in sleep assessment, determines that actigraphy is a useful tool to aid the study and clinical assessment of sleep disorders. This guideline was first published in 1995, with updates in 2002 and the most recent in 2007, which includes a new chapter on children and states that “actigraphy is indicated for delineating sleep patterns and documenting responses to treatment in infants and normal children and in special pediatric populations”, such as, for example, children with cystic fibrosis and psychiatric illnesses, such as Asperger’s syndrome [17]. Therefore, the last publication of the guide for the use of AASM actigraphy was considered to be the main inclusion criterion for studies. The exclusion criteria were defined with the objective of eliminating all studies that did not meet the research objective, especially those that did not carry out sleep assessments in children and adolescents with chronic conditions and/or their families.

Initially, the authors responsible for data collection revisited the review question and the objective of the integrative review. Subsequently, these authors were trained to use the data extraction template, which allowed for the comparison and organization of data, which were analyzed and grouped into categories.

The review of all the studies captured applying the search strategy was carried out by peers. Two of the authors, working independently, made a detailed reading of the titles and abstracts so as to ensure that the text addressed the guiding question and met the inclusion and exclusion criteria. In cases of doubt, we chose to include the publication and make the selection decision after reading the article in full.

In this integrative review, researchers ensured data collection reliability and validity through a comprehensive literature search using defined keywords and criteria. Multiple reviewers conducted a thorough quality assessment of the studies. We maintained transparency in our methods, documenting search strategies and decision-making processes for reproducibility. Discrepancies were resolved through consensus or third-party consultation, ensuring balanced data inclusion. The review process was dynamic, with regular updates incorporating the latest research. The interpretation of data, critical in maintaining the review’s precision and pertinence, was rigorously deliberated and collectively agreed upon by all contributing authors [18].

Descriptive data analysis was carried out using a chart created by the research group for data extraction and synthesis, containing the following information: article citation, country of origin, author’s field, objectives, subjects, study method and sleep assessment instruments used, level of evidence, and main findings. The chart made it possible to compare and organize the data, which were analyzed and grouped into categories [19]. The level of evidence was identified on the basis of the study design [20].

## 3. Results

**Figure 1:**
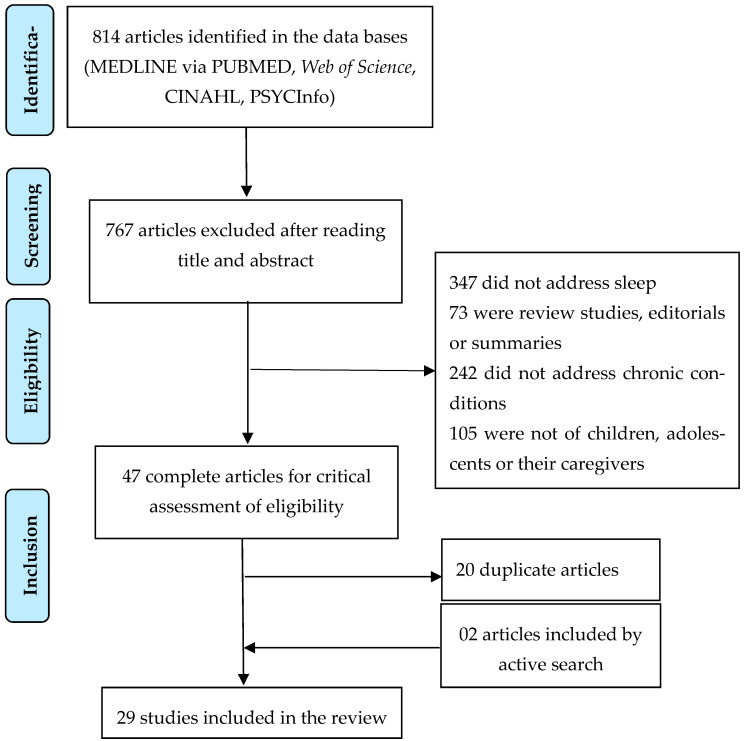
Flow diagram, based on Prisma (2020).

The database searches returned 814 references: 305 in MEDLINE^®^ via PubMed^®^, 115 in CINAHL, 259 in Web of Science and 135 in PSYCInfo. After the exclusion of 767 articles, 47 studies were selected to be read in full, of which 27 were retained in the results of this review. A further two articles were added by active search (Figure 1).

Studies were found in all years except 2021 and 2022. Prevalence was highest in 2019, when four studies were published [21,22,23,24].

In regard to the countries where studies were conducted, the United States (USA) predominated with 11 studies [25,26,27,28,29,30,31,32,33,34,35,36]. Of particular note were two multicenter studies, one in Brazil and the USA [37], and the other in Brazil, Portugal, and the USA [22].

Authors’ professional backgrounds were primarily in Medicine in 14 studies [23,24,27,29,31,33,36,38,39,40,41,42,43,44], followed by Nursing in six studies [22,30,34,37,45,46]. Interdisciplinary publications were also found, involving Nursing, Social Service and Medicine [21], Psychology and Medicine [28], Physiotherapy and Medicine [47], and Nursing and Medicine [35]. The study designs included 15 qualitative or descriptive studies [21,22,23,26,27,28,30,34,35,37,38,39,41,42,44,45,48], 13 case–control or cross-sectional studies [24,31,32,33,36,40,43,44,47,49,50] and one non-randomized controlled study [25,29]. The most common chronic condition was cancer, addressed in five studies [22,23,28,35,37], followed, in three studies each, by Duchenne muscular dystrophy [40,49,50], chronic kidney disease [24,29,33], asthma [30,31,43], and cystic fibrosis [27,42,47].

Regarding the participants of each study selected for the review, 14 conducted sleep assessments solely with children and/or adolescents with chronic conditions [22,24,25,26,29,30,31,33,34,35,36,37,38,40], 13 with family members [13,21,23,32,39,42,44,45,46,47,48,49,50], and 3 with both [27,41,43].

The instruments most used to assess sleep (Table 1) were the Pittsburgh sleep quality index (15 studies) [21,24,28,32,39,40,42,43,44,45,46,47,48,49,50], the sleep diary (5) [25,26,30,34,35,48], and actigraphy (5) [22,31,34,37,46].

Lastly, the studies selected were grouped empirically into four categories.

### 3.1. Category 1: Major Alterations in the Sleep Patterns of Children and Adolescents with Chronic Conditions

Eleven of the articles selected were included in this category.

One study of children (11 to 13 years old) with chronic diseases, such as asthma, epilepsy, and diabetes, showed that they had more trouble falling and staying asleep than healthy children [38].

The second study found that the sleep quality of children and adolescents (8 to 18 years old) with Duchenne muscular dystrophy (DMD) was significantly worse than healthy controls, particularly in regard to latency and duration [40].

One study of 301 children (from 1 to 16 years old) with mild and moderate chronic kidney disease (CKD) showed that 25% of them had moderate to severe sleep problems and that 29% reported having trouble sleeping or a lack of energy. Also, daytime sleepiness was a common complaint, especially from those with low glomerular function rate [33].

Another study showed that children (5 to 18 years old) with CKD returned higher scores for insomnia, daytime sleepiness and nocturnal waking [24]. Also, sleep disorders can be correlated with impaired health-related quality of life [29].

Children with allergic diseases (3 to 18 years old) also experienced various different sleep dysfunctions, the most common being problems falling/staying asleep and disorders of the transition from sleep to wakefulness [41].

No significant differences in sleep disorders were found in children with asthma (7 to 16 years old) when compared with healthy children [43]. However, when the asthma was not properly controlled, children from seven to nine years old showed less sleep efficiency and hygiene [31].

Another article identified disagreement between the reports of children with asthma (9 to 11 years old) and their parents; that is, when the parent said the child’s sleep quality was “excellent”, only a small percentage of the children agreed [30].

In a population of 91 children and adolescents (1 to 18 years old) with cystic fibrosis (CF), the most common sleep problems were long sleep latency, daytime sleepiness, and mouth breathing [27].

In children and adolescents (10 to 19 years old) with cancer undergoing chemotherapy treatment, sleep quality and hygiene were significantly worse. They also reported difficulty in falling and staying asleep and in going back to sleep [35].

All the studies included in this review showed impaired sleep patterns in children and adolescents with chronic conditions. The major alterations found were difficulty falling and staying asleep, worse sleep latency and duration, low energy, daytime sleepiness, insomnia, disorders of the transition from sleep to wakefulness, and worse sleep efficiency and hygiene.

### 3.2. Category 2: The Relationship between Sleep Disorders and Other Symptoms in Children and Adolescents with Chronic Conditions

This category comprised five studies.

Children and adolescents with chronic conditions experienced numerous signs and symptoms that can be intensified by poor sleep quality. Children from 8 to 16 years old with polyarticular arthritis, for example, reported that when sleep was impaired on the following day, their pain indices were higher [26].

Seven-day actigraphy in 17 adolescents (mean age 14 years) with chronic musculoskeletal pain showed that six slept between seven and eight hours, seven slept less than seven hours, and mean sleep efficiency was below 90% [34].

One comparative study using an actigraphic device in children and adolescents (8 to 18 years old) with cancer showed that, independent to the appearance of pain symptoms, these individuals did not sleep long enough [22].

Even after hospital discharge, alterations are still found in the sleep patterns of these children and adolescents with cancer (mean age 12 years). One study using actigraph data after discharge from hospital found sleep duration of approximately six hours and that the shorter this time, the greater the likelihood of problems with tiredness. Sleep interruptions of around one hour were found in the children and of two hours in the adolescents [37].

Another chronic condition causing sleep problems in children (6 to 12 years old) was Attention-Deficit Hyperactivity Disorder (ADHD). Sleep problems were significantly greater in children with symptoms of mood dysregulation and hyperactivity–impulsivity [36].

The studies included in this category showed a relationship between sleep disorders and other symptoms, in which individuals may experience intensified pain symptoms, elevated fatigue levels, and mood dysregulation problems.

### 3.3. Category 3: The Impaired Sleep Patterns of Families of Children and Adolescents with Chronic Conditions

Six of the articles selected were included in this category.

Overall, the families of children with chronic conditions reported worse sleep quality and more symptoms of insomnia and chronic sleep deprivation than families of healthy children [32] For instance, sleep disturbance was found in a study of parents of children (mean age 8.7 years) with respiratory disease and atopic dermatitis [32].

In the families of children with CF, poor sleep quality was found in most of the 23 mothers of children under 18 years old admitted to hospital [47].

One study using actigraph data showed that the families of technology-dependent children reported a mean 40 min less sleep per night than the families of healthy children and three times more sleep deprivation [46].

A study of the mothers of adolescents (mean age 18 years) with DMD found that they did not sleep as well as the mothers of healthy children, particularly in regard to sleep quality and latency. Specifically in the group of mother carers, worse sleep quality was found in those whose children had only recently begun using non-invasive ventilation [50].

Families in a study of children (3 to 12 years old) with acute lymphoblastic leukaemia also reported a mean of less than six hours of sleep per night, poor sleep efficiency, long sleep onset latencies, and sleepiness that impaired daytime functioning [28].

Another study also identified worse sleep hygiene in a group of 150 mothers of children (mean age 10 years) with epilepsy [21].

In this category, all the studies identified showed impairment to families’ sleep patterns, the significant disturbances being worse sleep latency, sleep hygiene and quality, insomnia, high rates of interrupted sleep, shorter sleep duration, and worse daytime functioning.

### 3.4. Category 4: Sleep Alterations and Their Relationship to Other Problems in the Families of Children and Adolescents with Chronic Conditions

Seven articles made up this category.

It has also been found that as sleep quality parameters deteriorate, so anxiety and depression increase, as seen in a study of mothers of children (mean age 8.1 years) with asthma and CF [42].

Of 66 families of children (mean age 10 years) with autism, 53% showed sleep disorders, including difficulty falling and staying asleep or early waking, which were related to worse moods [48].

The mothers were also found to have high cortisol levels, which correlated with impaired sexual function [49].

In another study, families of children under 19 years old with cancer reported sleep problems and anguish, showing that they were more likely to report parenting problems, chronic disease, insufficient social support, and daytime sleepiness [23].

The mothers in a study of children and adolescents (1 to 17 years old) with type 1 diabetes mellitus described heavier burdens of nocturnal care and sleep disorders, as well as fatigue, low energy, stress and irritability [39].

Mothers of children (four months to three years of age) with bronchopulmonary dysplasia reported sleeping a mean 5.8 h per night and taking a mean 37.8 min to initiate sleep, indicating disturbed sleep, which was detrimental to their quality of life [45].

A study of 308 families of children (0 to 12 years old) with epilepsy identified poor sleep quality. Caregiver anxiety, sleep quality, and the number of co-caregivers were predictors of caregiver depression [44].

The studies included in this category found sleep disorders in families to be associated with symptoms including anxiety, depression, worse mood, sexual dysfunction, anguish, fatigue, and stress.

## 4. Discussion

The review data highlight the fact that both children and adolescents with chronic conditions and their families display sleep disorders and that these have relationships with other symptoms or problems.

In childhood specifically, sleep is extremely important for development. In the early years of life, children spend more time sleeping than awake, making for rapid physical and cerebral development [68].

Sleep performs a restorative, energy-conservation, and protective function. Sleep deprivation can lead to significant alterations in the lives of the individuals concerned and their families, in regard to physical, occupational, cognitive and social performance, in addition to impairing the quality of care provided to children and adolescents with chronic conditions [69].

The findings of this review show that insufficient sleep predisposes individuals to physical and mental health problems, including diminished concentration, irritability, anxiety and depression, daytime sleepiness, anguish, fatigue and stress [70].

The Sociedade Portuguesa do Sono (2017) has recommended, by age group, that children from 0 to 3 months sleep 14 to 17 h a day; from 4 to 11 months, 12 to 15 h; one to two years, 11 to 14 h; three to five years, 10 to 13 h; six to thirteen years, 9 to 11 h; fourteen to seventeen years, 8 to 10 h; and, from eighteen years on, 7 to 9 hours [71].

Just as the amount of sleep is important, so too is its quality. Healthy sleep should be of an adequate duration, good quality, regular, and undisturbed [71].

Sleep is affected by intrinsic factors, such as sex, age, and puberty, as well as extrinsic factors, which include the environment, noise, school hours, technology use, bedtime, and even specific activities such as clinical care in hospital [70,72].

Alterations in a child’s health conditions may cause effects on their sleep quality [69]. In hospital, the major causes of sleep disturbances in children and their families are noise, excessive illumination, and the administration of medicines [68].

In sick children and adolescents, broken sleep can have quite significant effects on recovery, and it can also impair immune response and pain tolerance [73].

In addition to the characteristics of the chronic condition itself, other biological and treatment factors can influence the sleep pattern of these children and adolescents. A study carried out with 30 children with cystic fibrosis, between 6 and 18 years old, showed that 30% of them had Obstructive Sleep Apnea Syndrome, but also a high prevalence of Autonomic Nervous System disorders and nocturnal hypoxemia. Furthermore, a significant correlation was found between sleep disturbance and heart rate variability in patients with severe illness [74].

Although it was not a condition found in the studies selected for review, adenoid hypertrophy is a common chronic condition of the upper respiratory tract and one of the most common causes of airway obstruction in children, causing changes in mouth breathing, snoring, changes in voice, runny nose, and sleep apnea [75]. In a study carried out with children with hypertrophy of the adenoid system, it was demonstrated that they have a worse quality of life when compared to healthy children, mainly in terms of behavior and general perception of health and mental health [76].

Obstructive Sleep Apnea Syndrome (OSAS) associated with adenoid hypertrophy is one of the most prevalent sleep disorders in children. In a study with 61 caregivers of children with this condition, it was shown that OSAS has an important impact on the quality of these individuals’ lives, being considered large in 36% of participants, moderate in 31% and small in 33% [77].

The main instrument found in this review to evaluate the sleep pattern of children and adolescents with chronic conditions and their families was the Pittsburgh Sleep Quality Index (PSQI). However, polysomnography is still considered the gold standard method for sleep assessment [78]. Polysomnography is a technique that records multiple physiological variables throughout sleep time and is recommended by the American Academy of Sleep Medicine (AASM), when possible, for children with suspected Obstructive Sleep Apnea Syndrome, but also for the evaluation of sleep latency, sleep architecture, awakening rates, and sleep efficiency, among other specific events.

It is worth mentioning that some tests are considered basically necessary for carrying out polysomnography, namely electroencephalogram, electrooculogram and electromyogram, and that polysomnography lasts at least six hours. To evaluate sleep-disordered breathing, other parameters are considered, such as nasobuccal airflow, respiratory effort, oximetry and/or capnography, electrocardiogram, snoring sensor, body position sensor, and leg electromyogram [78].

Sleep restoration strategies include sleeping and waking at regular times; keeping the sleeping environment quiet, dark and at a comfortable temperature; disconnecting electronic appliances one hour before sleeping; avoiding stimulating food or drink before sleeping; and avoiding strenuous activities two hours before sleeping [71].

In hospital, strategies that can be used include dimming illumination and reducing noise at night, grouping health care procedures, and maintaining a homely environment, including having family members present [70].

Specifically in regard to the children’s families, some of the strategies that can be used involve reducing overload by sharing care; maintaining a support network that includes primary care services, school and church; and engaging with continued education provided by health professionals, who can inform families of treatment options, update their understanding and formulate a dynamic care plan, all in ways that fit in with the families’ routines [79].

It is known that nursing care brings countless advantages to the patient and their families, ranging from better acceptance of the disease, better adherence to treatment, control of symptoms and complications, clinical improvement, and quality of life, as well as greater participation and support from family members, minimizing the difficulties faced by everyone involved [80]. One of the main goals of pediatric nursing is to improve the quality of care for children and their families, promoting the health and wellbeing of the child [81]. Therefore, among health promotion actions, practices to guarantee an effective sleep pattern and, consequently, increase quality of life must be included in nursing care.

Research shows that children and teens with chronic conditions are more likely to experience sleep disorders. In the hospital environment, these problems are aggravated by the characteristics of the condition itself, and the type of treatment, infrastructure, and care provided by the healthcare team. Therefore, strategies to maintain a safe and calm environment, interact with children, and encourage self-care and group care, especially at night, must be part of the nursing process [2,81].

The present study demonstrates the presence of important sleep disorders in the population of children and adolescents with chronic conditions and their families. Furthermore, it has been discussed that there are numerous risks related to sleep deprivation, such as decreased cognitive performance, difficulty with memorization and learning, and diagnosis of other chronic conditions in adulthood, including diabetes and hypertension [82]. However, no study was found that used any strategy to manage and minimize this symptom, which is an important recommendation for future research.

As strategies to preserve and improve sleep patterns during hospitalization, non-pharmacological interventions that showed very positive effects were music therapy [70,83], storytelling [70], and massage [84], which favor the onset of sleep, in addition to increasing quality and duration.

A wide variety of chronic conditions were found in this review, stating that they all cause some harm to the sleep patterns of children and adolescents, such as difficulty in initiating and maintaining sleep [38], shorter duration [40], and worse latency [40]. In addition, other physical symptoms may increase, such as pain [26] and fatigue [37]. A similar result was found in the families of these children and adolescents, who also have worse latency [50] and insomnia [32], among others, in addition to the exacerbation of physical [39,49] and psychological symptoms [42,48]. Therefore, the results of this review prove that regardless of the chronic condition, these children and adolescents are more susceptible to developing sleep disorders. Therefore, sleep assessment is an important aspect to be considered during the physical examination of these individuals, so that management strategies for this symptom can be employed as early as possible, from the use of comfort measures to the use of non-pharmacological strategies.

Finally, even though the relevance of this topic is clearly demonstrated, the identification of many different instruments for assessing sleep in children and adolescents with chronic conditions and their families makes it difficult to compare the results.

## 5. Conclusions

The evidence revealed that the sleep patterns of children and adolescents with chronic conditions and their families are impaired, which can trigger or intensify physical and psychological symptoms. In the children’s families, impaired sleep was related to anxiety, depression, stress, and sexual dysfunction. It is necessary to implement specific strategies to normalize sleep patterns in this population, so as to foster improved quality of life for the children and their families.

The results of this review demonstrate that multidisciplinary care, including nursing practices, in addition to a holistic view of the subject and their family, are extremely important to meet the real needs of children and adolescents with chronic conditions. These individuals need specialized health care, with the aim of minimizing the effects of the condition itself, and hospitalization with treatment that enables healthy sleep patterns, which, consequently, improve the quality of life. Nurses need to plan and act effectively to reduce sleep disorders, based on the assessment of the sleep pattern of children with chronic conditions using valid and reliable instruments. For this, the professional needs to be aware of the structure and duration of sleep for each age group and, from there, be able to assess the causes of disturbances, acting to eliminate or minimize them. In addition, given the fragility and particularity of assistance to this population, attention also needs to be directed towards the family. Therefore, the nurse can guarantee care for children and adolescents and health education for the whole family, ensuring safe care and maintaining the quality of life in homes as well.

## Figures and Tables

**Table 1:** Characteristics of the sleep assessment instruments. Rio de Janeiro, 2023.

Instrument	Year/Country of Origin	Application
Pittsburgh Sleep Quality Index (PSQI)	1989/USA [51]	This instrument, comprising 19 questions relating to sleep quality and disturbance in the prior month, evaluates subjective sleep quality, sleep latency, sleep duration, sleep efficiency, sleep disorders, use of medication and daytime dysfunction. The questions are graded into scores from zero (no difficulty) to three (severe difficulty) and the sum total ranges from zero to 21, where the greater the value, the worse the sleep quality [51,52].
Wrist Actigraphy	1922/Germany [53].	The wrist actigraph is a device in the form of a wristwatch with the ability to store data over a specified time period. The system’s scoring programme can calculate sleep efficiency and duration. The device is based on an acceleration sensor, which converts external factors that produce motion and influence Circadian rhythm into measurements. The numerical representation is aggregated into a constant time interval, generally called a period (for example, one minute) [54,55].
CKiD Symptoms List	2003/USA and Canada [56]	Chronic Kidney Disease in Children (CKiD) is a prospective observational study of children and adolescents with mild and moderate renal insufficiency. It is a list of symptoms to be filled out for the prior month, indicating the number of days when the participant felt each of them, as well as describing the severity with which each symptom was experienced [56].In the study selected for this review, participants responded regarding the symptoms “weakness”, “early waking”, “falling asleep during the day” and “diminished alertness” [33].
Abbreviated Children’s Sleep Habits Questionnaire (CSHQ)	2000/USA [57]	This screening instrument answered by parents comprises 33 common items of child behaviour, grouped into three domains: dyssomnias, parasomnias and Respiratory Sleep Disturbances. The “long version” (48 items) includes other questions. Responses are scored on a three-point Likert scale (rarely = 0–1 night per week; sometimes = 2–4 nights per week; generally = 5–7 nights per week). Scores of 41 or more indicate possible sleep disorders [57,58].
Adolescent Sleep Wake Scale (ASWS)	2005/Italy and USA [59]	This was developed as a children’s sleep–wake scale and is applied to adolescents from 12 to 18 years old. It comprises 28 items, divided into five dimensions of behaviour—going to bed, falling asleep, maintaining sleep, reinitiating sleep, and returning to wakefulness—and uses a six-point scale (“always”, “often, if not always”, “often”, “sometimes”, “now and then” and “never”). The higher the score, the better the sleep quality [59].
Adolescent Sleep Hygiene Scale	2005/Italy and USA [59]	This is an adaptation of the Children’s Sleep Hygiene Scale, applicable to adolescents from 12 to 18 years old. It contains 28 items that assess issues that facilitate and hinder sleep, which are divided into nine domains: physiological, cognitive, emotional, sound environment, daytime sleep, substances, sleep routine, sleep stability, and room/bed sharing. It uses a six-point scale (“always”, “often, if not always”, “quite often”, “sometimes”, “now and then” and “never”), where the higher the score, the better the sleep-hygiene behaviour [59].
Epworth Sleepiness Scale	1991/Australia [60]	This self-applied questionnaire assesses the likelihood of falling asleep (never, slight chance, moderate chance and high chance) in eight activities of daily living: sitting and reading; watching TV; sitting inactive in a public place; as a passenger in a car for an hour or more without stopping for a break; lying down to rest when circumstances permit; sitting and talking to someone; sitting quietly after a meal without alcohol; and in a car, while stopping for a few minutes in traffic or at a traffic light. Total scores range from 0 to 24. A score of more than 10 suggests a diagnosis of excessive daytime sleepiness [60,61].
Paediatric Sleep Questionnaire	2000/USA [62]	Comprises around 20 items, which can be answered in about 5 min. The questionnaire, which evaluates symptoms of sleep disorders in children, includes scales for obstructive respiratory sleep-related disorders (mouth breathing, night sweats, nocturia, enuresis, nasal congestion, sleep bruxism, retarded growth, obesity), snoring, daytime sleepiness, insomnia and restless leg syndrome. Scores above 0.33 are considered to suggest a diagnosis of sleep-related disorder [62].
Sleep Disturbance Scale for Children	1996/Italy [63]	Consists of a 26-item sleep evaluation instrument applicable to children from 3 to 18 years old. It assesses disorders of initiating and maintaining sleep, sleep breathing, arousal, sleep–wake transition, excessive daytime sleepiness, and sleep hyperhidrosis. It uses a Likert-type scale scoring from 26 to 130. A score of 39 was established as the cut-off point suggestive of sleep disorders [63].
Children’s Sleep Hygiene Scale (CSHS)	2002/USA [64]	This examines children’s sleep hygiene as reported by parents. It consists of 22 items that assess physiological, cognitive, emotional, environmental, sleep routine, and sleep-stability issues associated with sleep hygiene. Responses are on a six-point Likert scale ranging from Never to Always, with higher scores indicating better sleep hygiene [64].
General Sleep Inventory	2009/USA [65]	This scale uses parent-report data to assess whether the child has a regular bedtime and, if so, at what time, and whether the family has enforced that bedtime in the prior five nights, and whether the family has one or more sleep routines and, if so, whether the family has followed those routines in the prior five nights. It also evaluates indicators of sleep routines: interaction with parents, non-interaction with parents, watching television or video, or eating a snack, and hygiene-related behaviour. It uses a six-point Likert scale from Never to Always [65].The study selected for this review also evaluated whether the children shared a room, shared a bed, slept in their own bed, or in another room, and how often they were disturbed by domestic and/or neighbourhood noise, as well as how many people lived in the domicile [31].
Insomnia Severity Index (ISI)	1993/USA [66]	This instrument evaluates patients’ perceptions of their insomnia. There are three versions: patient (self-report), third-party (spouse, for example), and doctor. It consists of seven items that assess severity of difficulty initiating and maintaining sleep, early morning waking, satisfaction with the sleep pattern, interference in daytime functioning, perception of impairment attributed to sleep problem and degree of distress or concern caused by the sleep problem. Responses are on a scale of 0 to 4 points and the total ranges from 0 to 28. The higher the score, the more suggestive of severe insomnia [66,67].

## Data Availability

The data presented in this study are available in the article.
